# Comparative Outcomes of Day 3 and Day 5 Frozen–Thawed Embryo Transfers: A Retrospective Clinical Study

**DOI:** 10.3390/biomedicines13102412

**Published:** 2025-10-01

**Authors:** Fatma Kılıç Hamzaoğlu, Serdar Dilbaz, Runa Özelçi, Onur Kaya, Emine Utlu Özen

**Affiliations:** 1 Department of Gynecology and Obstetrics, Faculty of Medicine, Necmettin Erbakan University, Konya 42090, Turkey; 2 Department of Gynecology and Obstetrics, University of Health Sciences, Etlik City Hospital, Ankara 06170, Turkey; prof@serdardilbaz.com (S.D.); runakara@gmail.com (R.Ö.); onurkaya@gmail.com (O.K.); emineutlu@gmail.com (E.U.Ö.)

**Keywords:** frozen–thawed embryo transfer, cleavage-stage embryo, blastocyst-stage embryo

## Abstract

**Aim**: This study aimed to compare the clinical pregnancy outcomes of Day 3 (cleavage-stage) and Day 5 (blastocyst) frozen–thawed embryo transfers in assisted reproductive technology (ART) cycles. **Methods**: A retrospective analysis was conducted on 6455 patients who underwent frozen–thawed embryo transfer (FET) between 1 April 2010 and 1 April 2023, at SBÜ Etlik Zübeyde Hanım Women’s Health Training and Research Hospital. Patients were categorized based on embryo transfer type, and clinical outcomes were compared using statistical analyses. **Results**: Pregnancy rates were significantly higher in the blastocyst transfer group (*p* = 0.031). The cleavage-stage group had a higher number of embryo transfers (*p* < 0.001), likely due to lower implantation potential per embryo. Patients with diminished ovarian reserve had lower embryo freezing rates (*p* < 0.001). Blastocyst-stage transfers showed better synchronization with endometrial receptivity but carried a risk of embryo attrition. **Conclusions**: Blastocyst transfer is associated with higher pregnancy rates and should be preferred when viable embryos are available. However, cleavage-stage transfer remains an alternative for patients with poor embryo development. An individualized approach is essential for optimizing ART outcomes.

## 1. Introduction

In recent years, the prevalence of infertility among couples has been increasing, leading a significant number of couples to seek infertility treatment. Assisted reproductive technologies (ART) are employed to achieve pregnancy in patients who cannot conceive through medical or surgical methods [1]. In patients undergoing frozen–thawed embryo transfer (FET), various factors such as the day of embryo freezing, the number and quality of transferred embryos, maternal age, endometrial thickness, and the vitrification method used have been reported to affect embryo viability after thawing and clinical pregnancy outcomes [2].

Blastocyst culture allows for an extended observation of embryo development potential, thereby improving embryo-endometrium synchronization. These culture systems and blastocyst transfer applications provide not only improved pregnancy outcomes but also a more physiological option that reduces the risks and complications associated with multiple pregnancies [3].

A randomized study reported that although pregnancy rates following blastocyst embryo transfer were higher after vitrification and thawing compared to cleavage-stage embryo transfer, there was no statistically significant difference between the two groups [4,5]. Studies on embryo transfer timing have suggested that selecting the best morphology embryo at the cleavage (Day 3) or blastocyst (Day 5) stage can yield pregnancy rates of up to 50% [3,6].

A study by Levens et al. found that implantation rates were higher following the transfer of thawed Day 5 blastocysts compared to Day 6 blastocysts, but there was no significant difference in clinical pregnancy rates between the two groups [7]. Additionally, in a prospective randomized controlled study involving fresh embryo transfers, Day 3 cleavage-stage embryos had a higher reported pregnancy success rate compared to previous studies assessing Day 5 blastocyst transfers [8].

However, there is a noticeable lack of sufficient studies comparing Day 3 cleavage-stage and Day 5 blastocyst frozen embryo transfers. This study aims to analyze the outcomes of Day 3 and Day 5 embryo transfers following the freezing-thawing process at our clinic.

This study aims to investigate the clinical pregnancy outcomes of Day 3 (cleavage-stage) and Day 5 (blastocyst) frozen–thawed embryo transfers. The goal is to determine whether transferring embryos at different developmental stages significantly influences pregnancy success rates. By identifying the factors contributing to successful implantation and clinical pregnancy, this study seeks to provide evidence-based insights that can enhance embryo transfer protocols and improve patient outcomes in assisted reproductive treatments.

Optimizing embryo transfer strategies is crucial in improving success rates in assisted reproductive technologies. Although both cleavage-stage and blastocyst-stage transfers are widely used, there is no definitive consensus on which approach yields better clinical outcomes in frozen–thawed embryo transfer cycles. By comparing these two transfer strategies, this study aims to fill a critical gap in the literature, offering valuable data for reproductive specialists. The findings will contribute to refining clinical decision-making processes, improving individualized treatment plans, and ultimately increasing the chances of successful pregnancies for patients undergoing infertility treatments.

## 2. Materials and Methods

This study is a retrospective clinical study conducted at the Assisted Reproductive Technology (ART) Unit of SBÜ Etlik Zübeyde Hanım Women’s Health Training and Research Hospital. Data from patients who underwent frozen–thawed embryo transfer (FET) between 1 April 2010, and 1 April 2023, were retrospectively analyzed. The study included female patients under the age of 40 who underwent frozen–thawed embryo transfer at the cleavage-stage (Day 3) or blastocyst-stage (Day 5) during the specified period. Only patients who had a single embryo transfer were included to maintain homogeneity in clinical outcomes. Patients aged 40 years or older, those who did not have viable embryos for freezing, those who underwent fresh embryo transfer, those who had multiple embryo transfers in a single cycle, and those with incomplete medical records or lost to follow-up were excluded from the study.

Male factor infertility was not an exclusion criterion; infertility etiology including male factor was recorded. Because this is a retrospective study, potential selection and information bias cannot be completely ruled out and were acknowledged as study limitations. The study period spanned 13 years; although no major changes occurred in ovarian stimulation protocols, cryopreservation, or thawing techniques at the center, minor refinements were implemented as part of routine practice. Overall, both stimulation and vitrification protocols remained almost unchanged throughout the study period, ensuring comparability of outcomes across the years. This factor was considered in the discussion. Due to the retrospective design, no formal power analysis was conducted; instead, all eligible cases during the study period were included. The relatively small number of cleavage-stage transfers is acknowledged as a limitation.

Patient medical records were reviewed retrospectively to extract demographic characteristics, infertility history, ovarian reserve markers such as follicle-stimulating hormone (FSH) levels and antral follicle count (AFC), cycle characteristics, endometrial thickness, embryo quality, and pregnancy outcomes. The clinical parameters analyzed included maternal age, body mass index (BMI), infertility duration and etiology, number of stimulation cycles, endometrial thickness on embryo transfer day, embryo transfer type, and pregnancy outcomes such as positive β-hCG, clinical pregnancy, and implantation rate.

Embryos were cryopreserved using vitrification techniques, which involved the use of cryoprotectants followed by ultra-rapid freezing in liquid nitrogen. Thawing was performed using a stepwise dilution of cryoprotectants to ensure embryo viability, and post-thaw survival rates were recorded. Endometrial preparation for embryo transfer was conducted using either a natural cycle or hormone replacement therapy (HRT). In the natural cycle group, ovulation was monitored through serum estradiol and luteinizing hormone (LH) measurements, while in the HRT group, estrogen and progesterone were administered to achieve optimal endometrial thickness. Embryo transfer was performed using a soft catheter under ultrasound guidance, and luteal phase support was provided with progesterone supplementation.

The primary outcome measure of the study was clinical pregnancy, defined as the presence of a gestational sac with a fetal heartbeat confirmed by ultrasound. Secondary outcomes included implantation rate, biochemical pregnancy rate, and miscarriage rate. Missing data were minimal; cases with incomplete essential variables were excluded from analysis.

### Statistical Analysis

Statistical analyses were performed using SPSS (Statistical Package for Social Science) version 22. The normality of continuous variables was assessed using the Kolmogorov–Smirnov and Shapiro–Wilk tests. Descriptive statistics were presented as means and standard deviations for continuous variables and percentages for categorical variables. Comparisons between Day 3 and Day 5 embryo transfer groups were conducted using the chi-square test for categorical variables, the Mann–Whitney U test for continuous variables that were not normally distributed, and the independent *t*-test for normally distributed continuous variables. Receiver Operating Characteristic (ROC) analysis was performed to evaluate the diagnostic performance of clinical parameters in predicting transfer type, and ROC curves along with the area under the curve (AUC) were analyzed. Sensitivity and specificity values along with their 95% confidence intervals were calculated for the optimal cut-off points.

For continuous numerical variables influencing transfer type, univariate logistic regression analysis was conducted using the Enter method. Odds ratios (OR) and their 95% confidence intervals were calculated. A *p*-value of less than 0.05 was considered statistically significant.

## 3. Results

The study was completed with 6455 patients undergoing IVF treatment. When patients were evaluated based on embryo freezing characteristics, there was no significant difference in age and BMI. However, the rate of embryo freezing was lower in patients with diminished ovarian reserve and age-related infertility, whereas it was higher in cases of unexplained infertility (*p* < 0.001). Additionally, patients who underwent embryo freezing had a higher prevalence of comorbidities and PCOS (*p* = 0.025 and *p* < 0.001, respectively) (Table 1).

When patients were evaluated based on embryo freezing characteristics, no significant difference was observed in endometrial thickness on the day of embryo transfer and IVF treatment outcomes. However, the number of cycles, duration of infertility, AFC, and number of embryo transfers were significantly higher in patients who underwent embryo freezing (*p* < 0.001, *p* = 0.009, *p* < 0.001, and *p* < 0.001, respectively). On the other hand, FSH levels were significantly lower in patients who underwent embryo freezing (*p* < 0.001) (Table 2).

Among the 349 patients who underwent embryo freezing, 301 had blastocyst transfer, 34 had cleavage-stage transfer, and 14 had embryo transfer through other methods. When patients were evaluated based on embryo transfer type, there was no significant difference in age. However, the rate of blastocyst transfer was lower in patients with hormonal ovulatory disorders and pelvic adhesions (*p* < 0.001 and *p* = 0.003, respectively). Additionally, patients who underwent blastocyst transfer had higher BMI and comorbidity rates (*p* = 0.040 and *p* = 0.023, respectively), while the rate of PCOS was lower (*p* < 0.001) (Table 3).

When patients were evaluated based on embryo transfer type, there was no significant difference in the number of cycles, duration of infertility, FSH, AFC, and endometrial thickness on the day of embryo transfer. However, the number of embryo transfers was significantly higher in patients who underwent cleavage-stage transfer (*p* < 0.001), while the pregnancy rate was significantly higher in patients who underwent blastocyst transfer (*p* = 0.031) (Table 4).

Age, BMI, number of cycles, duration of infertility, FSH, AFC, number of embryo transfers, and endometrial thickness on the day of embryo transfer did not show statistically significant diagnostic performance in predicting embryo transfer type. However, the number of embryo transfers showed statistically significant diagnostic performance (*p* = 0.045) (Table 5).

Each unit increase in the number of embryo transfers statistically significantly increased the likelihood of blastocyst transfer by 1.314 times (*p* < 0.001) (Table 6).

## 4. Discussion

The findings of this study contribute to the ongoing debate regarding the optimal timing of embryo transfer in frozen–thawed cycles. The comparison of Day 3 (cleavage-stage) and Day 5 (blastocyst) embryo transfers revealed significant differences in pregnancy success rates, aligning with existing literature that suggests blastocyst transfer may offer advantages in implantation potential and clinical pregnancy rates [3]. Our study found that pregnancy rates were significantly higher in the blastocyst transfer group compared to the cleavage-stage transfer group, consistent with prior research indicating improved implantation success with blastocyst-stage embryos [4,6]. However, some studies have reported no significant difference in cumulative live birth rates between cleavage-stage and blastocyst-stage transfers, suggesting that embryo transfer timing may not be the sole determinant of success [5].

Our findings that Day 5 transfer is associated with higher clinical pregnancy rates are broadly consistent with retrospective comparisons reporting superior outcomes with blastocyst-stage transfer in FET cycles, although prognosis-based selection remains a plausible alternative explanation for at least part of the effect [9,10,11]. Notably, cohorts in which Day 5 was preferentially offered to patients with more available embryos or better embryo development trajectories also observed higher success after blastocyst transfer, underscoring the need for cautious interpretation and adjusted analyses [9,10].

The timing of blastocyst formation further refines expectations. Beyond the Day-5 versus Day-6 contrast discussed earlier, very late blastulation (Day 7) may still yield acceptable outcomes in selected cases, but with attenuated success compared with earlier blastocysts [12]. In centers adopting sequential strategies within FET cycles (e.g., staged or sequential transfer policies), retrospective data indicate that individualized planning can maintain reasonable outcomes while accommodating variable embryo development dynamics [13]. These observations align with our conclusion that Day 3 transfer remains a pragmatic alternative when extended culture carries a high risk of attrition, particularly in poor responders.

When contextualizing our results within broader ART practice, comparisons of fresh versus frozen–thawed transfers have shown that FET can achieve at least comparable, and in many contexts improved, clinical outcomes—likely related to endometrial synchronization and refinements in vitrification [14]. This backdrop supports our reliance on FET and provides biological plausibility for the synchronization advantage we observed with Day 5 transfers.

Another clinically relevant scenario is the transfer of blastocysts derived from previously frozen–thawed cleavage embryos. Real-world and propensity-matched cohorts have reported reassuring pregnancy outcomes after such transfers, suggesting that clinics can safely freeze on Day 3 and defer selection to the blastocyst stage when embryo numbers are limited at the first decision point [10]. More recent retrospective data comparing blastocysts originating from fresh versus previously frozen cleavage embryos similarly support the feasibility of these pathways without clear compromise in key outcomes, particularly in carefully selected patients [15]. These findings align with our recommendation to individualize strategy according to embryo availability and development potential.

Finally, single embryo transfer (SET) policies in frozen–thawed blastocyst cycles have been associated with robust pregnancy rates alongside marked reductions in multiple gestation risk, supporting our practice of SET to optimize maternal–fetal safety [11]. Together, these lines of evidence reinforce a pragmatic algorithm: prefer Day 5 transfer when adequate embryo numbers allow safe extended culture; consider Day 3 transfer in poor-prognosis scenarios to avoid total attrition; and implement SET whenever feasible to reduce multiples while maintaining high clinical effectiveness [9,10,11,12,13,14,15].

One of the key considerations in choosing between cleavage-stage and blastocyst-stage transfers is the synchronization between embryo development and endometrial receptivity. Blastocyst-stage embryos are thought to provide better alignment with endometrial receptivity, potentially enhancing implantation outcomes [3]. The improved synchronization may explain the higher pregnancy rates observed in the blastocyst transfer group in our study. Additionally, vitrification techniques have been shown to maintain high survival rates for embryos post-thawing, making blastocyst transfer a feasible and effective strategy [2]. Despite these advantages, our findings also indicate that patients undergoing cleavage-stage transfer had a higher number of embryo transfers, which may suggest a compensatory approach due to lower implantation potential per embryo.

However, extending embryo culture to the blastocyst stage is not without risks. Our study found that patients with hormonal ovulatory disorders and pelvic adhesions had lower blastocyst transfer rates, which may be attributed to lower embryo development potential in these subgroups. Previous studies have indicated that extended embryo culture can lead to increased embryo attrition, potentially reducing the number of viable embryos available for transfer or freezing [3,16]. This is particularly relevant for patients with diminished ovarian reserve or poor embryo quality, where Day 3 transfers may be a more viable option to ensure an embryo is available for transfer [17]. In our study, we observed that the rate of embryo freezing was lower in patients with diminished ovarian reserve and age-related infertility, which aligns with findings that these patients often produce fewer viable embryos capable of reaching the blastocyst stage [7,8].

Another factor influencing transfer outcomes is the role of delayed blastocyst formation. Studies have suggested that blastocysts that develop on Day 6 or later have lower implantation potential compared to those that reach the blastocyst stage by Day 5 [18,19]. Our study did not specifically analyze Day 6 blastocysts, but the higher implantation and pregnancy rates in the Day 5 blastocyst group suggest that timely blastocyst development may be a key predictor of success. Additionally, stress management has been recognized as an important factor influencing ART outcomes, with research suggesting that psychological well-being can impact implantation and pregnancy rates [1]. While our study did not assess stress levels, it remains an important consideration in optimizing embryo transfer outcomes.

Our study also found that embryo transfer type was not significantly associated with maternal age, BMI, infertility duration, or endometrial thickness on the day of transfer. This is consistent with findings from previous studies that have suggested that these parameters alone may not be sufficient predictors of embryo transfer success [5,6]. However, our statistical analysis revealed that the number of embryo transfers was a significant predictor of blastocyst transfer likelihood, indicating that patients who underwent more embryo transfers were more likely to receive blastocyst-stage embryos. This suggests that repeated transfer attempts may be associated with better embryo selection and improved outcomes over time.

Overall, our findings support the notion that blastocyst transfer is associated with higher implantation and pregnancy rates, making it a preferred option when sufficient embryos are available for extended culture. However, cleavage-stage transfer remains a valuable alternative in cases where blastocyst development is uncertain or where immediate transfer is necessary due to patient-specific factors. Further prospective studies with long-term follow-up data are needed to confirm the benefits and limitations of each transfer strategy and to refine patient selection criteria for optimal ART outcomes.

### Limitations

This study has several limitations. First, its retrospective design inherently carries a risk of selection and information bias. Second, the study groups were unbalanced, with a relatively small number of Day 3 transfers compared to Day 5, which may weaken the statistical power of some comparisons. Third, although the study period spanned 13 years, stimulation and vitrification protocols remained almost unchanged, minimizing temporal variability. Nevertheless, incremental improvements in laboratory conditions and culture systems could have influenced the outcomes. Fourth, male factor infertility, although included in the cohort, was not analyzed in detail, and therefore its potential impact on embryo quality and transfer success could not be fully assessed. Finally, due to the retrospective design, long-term outcomes such as live birth rates and neonatal health were not available.

## 5. Conclusions

In conclusion, this study provides valuable insights into the comparative outcomes of Day 3 and Day 5 frozen–thawed embryo transfers, highlighting the advantages of blastocyst transfer in achieving higher pregnancy rates. The findings support the preference for blastocyst transfer when a sufficient number of viable embryos are available, as it offers better synchronization with endometrial receptivity and improved implantation potential. However, cleavage-stage transfer remains a viable option for patients with diminished ovarian reserve or poor embryo development capacity. Given the complexities associated with embryo development, implantation, and patient-specific factors, an individualized approach to embryo transfer remains essential. Future large-scale prospective studies are needed to further validate these findings and optimize ART protocols to enhance clinical pregnancy outcomes.

## Figures and Tables

**Table 1:** Evaluation of Patients’ Clinical Characteristics According to Embryo Cryopreservation Status.

	Embryo Freezing		
	No (*n* = 6106)	Yes (*n* = 349)	*p* *
Age	32 ± 6	31 ± 5	0.083 *
BMI	26.3 ± 4.9	26.1 ± 4.8	0.208 *
Cause of Infertility			
Male factor	2335 (38.2)	139 (39.8)	0.555 **
Tubal factors	374 (6.1)	29 (8.3)	0.101 **
Diminished ovarian reserve	1019 (38.6)	29 (14.5)	<0.001 **
Age-related	661 (10.8)	18 (5.2)	<0.001 **
Hormono-ovulatory disorder	1097 (18.0)	70 (20.1)	0.323 **
Unexplained infertility	1750 (28.7)	142 (40.7)	<0.001 **
Pelvic adhesions	47 (0.8)	1 (0.3)	0.307 **
Comorbidity			
Present	27.4	30.2	0.025 **
Absent	72.6	69.8	
PCOS			
Present	680 (11.2)	80 (23.0)	<0.001 **
Absent	5418 (88.8)	268 (77.0)	

* Mann–Whitney U test; ** Chi-square test.

**Table 2:** Evaluation of Patients’ IVF Treatment and Pregnancy Characteristics According to Embryo Cryopreservation Status.

	Embryo Freezing		
	No (*n* = 6106)	Yes (*n* = 349)	*p*
Number of cycles	2 ± 1	3 ± 1	<0.001 *
Duration of infertility (months)	72 ± 54	76 ± 49	0.009 *
FSH	8.5 ± 12.2	6.7 ± 2.9	<0.001 *
AFC	12 ± 8	17 ± 9	<0.001 *
Number of embryo transfers	1 ± 1	2 ± 1	<0.001 *
Endometrial thickness on embryo transfer day	10.35 ± 3.13	10.16 ± 2.09	0.385 *
IVF treatment outcome			
Pregnant	577 (9.5)	43 (12.5)	0.063 **
Not Pregnant	5501 (90.5)	300 (87.5)	

* Mann–Whitney U test; ** Chi-square test.

**Table 3:** Evaluation of Patients’ Clinical Characteristics According to Type of Embryo Transfer.

	Blastocyst Transfer (*n* = 301)	Cleavage-Stage Transfer (*n* = 34)	*p*
Age	31 ± 5	31 ± 4	0.750 *
BMI	26.3 ± 4.9	24.3 ± 3.0	0.040 *
Cause of infertility			
Male factor	116 (38.5)	17 (50.0)	0.195 **
Tubal factors	26 (8.6)	2 (5.9)	0.582 **
Diminished ovarian reserve	29 (14.9)	0 (0)	0.951 **
Age-related	17 (5.6)	1 (2.9)	0.507 **
Hormono-ovulatory disorder	51 (16.9)	17 (50.0)	<0.001 **
Unexplained infertility	123 (40.9)	12 (35.3)	0.530 **
Pelvic adhesions	0 (0)	1 (2.9)	0.003 **
Comorbidity			
Present	30.8	23.4	0.023 **
Absent	69.2	76.6	
PCOS			
Present	61 (20.3)	17 (50.0)	<0.001 **
Absent	239 (79.7)	17 (50.0)	

* Mann–Whitney U test; ** Chi-square test.

**Table 4:** Evaluation of Patients’ IVF Treatment and Pregnancy Characteristics According to Type of Embryo Transfer.

	Blastocyst Transfer (*n* = 301)	Cleavage-Stage Embryo Transfer (*n* = 34)	*p*
Number of cycles	3 ± 1	2 ± 1	0.468 *
Duration of infertility (months)	74 ± 47	78 ± 47	0.518 *
FSH	6.72 ± 2.69	7.35 ± 4.81	0.724 *
AFC	17 ± 9	18 ± 9	0.376 *
Number of embryo transfers	1 ± 0	2 ± 1	<0.001 *
Endometrial thickness on embryo transfer day	10.19 ± 2.13	9.72 ± 1.65	0.261 *
IVF treatment outcome			
Pregnant	42 (14.2)	1 (2.9)	0.031 **
Not Pregnant	253 (85.8)	33 (97.1)	

* Mann–Whitney U test; ** Chi-square test.

**Table 5:** ROC Analysis of Patients’ Clinical and IVF Treatment Characteristics for Predicting the Type of Embryo Transfer.

	AUC (CI)	*p*	Cut-off Value	Sensitivity (%)	Specificity (%)
Age	0.481 (0.379–0.583)	0.780	30.5	50.0	55.0
BMI	0.578 (0.471–0.685)	0.246	24.85	53.1	50.0
Number of cycles	0.549 (0.419–0.678)	0.469	2.5	35.8	30.0
Duration of infertility (months)	0.483 (0.347–0.619)	0.805	63.0	45.4	45.0
FSH	0.530 (0.386–0.673)	0.658	6.18	50.4	50.0
AFC	0.424 (0.279–0.550)	0.255	16.5	41.5	55.0
Number of embryo transfers	0.369 (0.223–0.515)	0.045	1.5	31.2	50.0
Endometrial thickness on embryo transfer day	0.601 (0.488–0.714)	0.131	9.35	60.4	40.0

Area under the curve (AUC), Confidence interval (CI).

**Table 6:** IVF Treatment Outcome According to Patients’ Age, FSH, Pronuclear Count, Number of Grade 1 Embryos, and Number of Grade 3 Embryos.

	**Type of Embryo Transfer**	***p***
Number of embryo transfers OR (%95 GA)	1.314 (0.661–1.981)	<0.001 *

* Univariate logistic regression analysis was used.

## Data Availability

The data that support the findings is contained within the article.

## References

[1] Koumparou M., Bakas P., Pantos K., Economou M., Chrousos G. (2021). Stress management and In Vitro Fertilization (IVF): A pilot randomized controlled trial. Psychiatriki.

[2] Loutradi K.E., Kolibianakis E.M., Venetis C.A., Papanikolaou E.G., Pados G., Bontis I., Tarlatzis B.C. (2008). Cryopreservation of human embryos by vitrification or slow freezing: A systematic review and meta-analysis. Fertil. Steril..

[3] Glujovsky D., Retamar A.M.Q., Sedo C.R.A., Ciapponi A., Cornelisse S., Blake D. (2022). Cleavage-stage versus blastocyst-stage embryo transfer in assisted reproductive technology. Cochrane Database Syst. Rev..

[4] Fernández-Shaw S., Cercas R., Bra-a C., Villas C., Pons I. (2015). Ongoing and cumulative pregnancy rate after cleavage-stage versus blastocyst-stage embryo transfer using vitrification for cryopreservation: Impact of age on the results. J. Assist. Reprod. Genet..

[5] De Croo I., Colman R., De Sutter P., Stoop D., Tilleman K. (2022). No difference in cumulative live birth rates between cleavage versus blastocyst transfer in patients with four or fewer zygotes: Results from a retrospective study. Human Reprod. Open.

[6] Schwärzler P., Zech H., Auer M., Pfau K., Göbel G., Vanderzwalmen P., Zech N. (2004). Pregnancy outcome after blastocyst transfer as compared to early cleavage stage embryo transfer. Hum. Reprod..

[7] Levens E., Whitcomb B., Hennessy S., James A., Yauger B., Larsen F. (2008). Blastocyst development rate impacts outcome in cryopreserved blastocyst transfer cycles. Fertil. Steril..

[8] Scholtes M.C., Zeilmaker G.H. (1996). A prospective, randomized study of embryo transfer results after 3 or 5 days of embryo culture in in-vitro fertilization. Fertil. Steril..

[9] Rao J., Qiu F., Tian S., Yu Y., Zhang Y., Gu Z., Cai Y., Jin F., Jin M. (2021). Clinical outcomes for Day 3 double cleavage-stage embryo transfers versus Day 5 or 6 single blastocyst transfer in frozen–thawed cycles: A retrospective comparative analysis. J. Int. Med. Res..

[10] Xiong F., Li G., Sun Q., Wang S., Wan C., Chen P., Yao Z., Zhong H., Zeng Y. (2019). Clinical outcomes after transfer of blastocysts derived from frozen–thawed cleavage embryos: A retrospective propensity-matched cohort study. Arch. Gynecol. Obstet..

[11] Hur Y.S., Ryu E.K., Song S.H., Yoon S.H., Lim K.S., Lee W.D., Lim J.H. (2016). A retrospective study of single frozen-thawed blastocyst transfer. Clin. Exp. Reprod. Med..

[12] Liu X., Lou H., Zhang J., Du M., Du Y., Wu S., Guan Y., Liu J. (2022). Clinical outcome analysis of frozen-thawed embryo transfer on day 7. Front. Endocrinol..

[13] Zhou W., Cheng Z., Wang C., Feng Y. (2023). Clinical outcome analysis of sequential transplantation of frozen-thawed embryo transfer cycle: A retrospective study. Medicine.

[14] Shen C., Shu D., Zhao X., Gao Y. (2014). Comparison of clinical outcomes between fresh embryo transfers and frozen-thawed embryo transfers. Iran. J. Reprod. Med..

[15] Tran H.P., Hoang T.T.D., Tran H.L.B., Huynh T.N.K., Do V.N.T., Mai C.K., Dang S.T. (2025). Pregnancy Outcomes of Frozen-Thawed Blastocysts versus Blastocysts Derived from Frozen-Thawed Cleavage Embryos: A Retrospective Study. Int. J. Fertil. Steril..

[16] Sunkara S.K., Siozos A., Bolton V.N., Khalaf Y., Braude P.R., El-Toukhy T. (2010). The influence of delayed blastocyst formation on the outcome of frozen-thawed blastocyst transfer: A systematic review and meta-analysis. Human. Reprod..

[17] Carvalho B.R., Barbosa M.W., Bonesi H., Gomes Sobrinho D.B., Cabral Í.O., Barbosa A.C., Silva A.A., Iglesias J.R., Nakagawa H.M. (2017). Embryo stage of development is not decisive for reproductive outcomes in frozen-thawed embryo transfer cycles. JBRA Assist. Reprod..

[18] Corti L., Cermisoni G.C., Alteri A., Pagliardini L., Ambrosini G., Andrisani A., Papaleo E., Viganò P., Noventa M. (2022). Clinical outcomes deriving from transfer of blastocysts developed in day 7: A systematic review and meta-analysis of frozen-thawed IVF cycles. Reprod. Sci..

[19] Dai X., Gao T., Xia X., Cao F., Yu C., Li T., Li L., Wang Y., Chen L. (2021). Analysis of biochemical and clinical pregnancy loss between frozen-thawed embryo transfer of blastocysts and day 3 cleavage embryos in young women: A comprehensive comparison. Front. Endocrinol..

